# Supplementary material for: Wolbachia endosymbionts in two Anopheles species indicates independent acquisitions and lack of prophage elements
Source: Microb Genom. 2022 Apr 21;8(4):000805. doi: 10.1099/mgen.0.000805 (PMC9453072; doi:10.1099/mgen.0.000805)
Supplement: Supplementary material 1 [file mgen-8-0805-s001.pdf]

Supplementary figure 1: Comparison of read depths on different genomes. Each panel shows genome alignments as depicted in Figure 5 using the Blast Ring Image Generator (BRIG, [1]), with an additional outer ring that shows the depth of mapping of reads from various sources, in log-2 space. The rings on different panels are not to scale. Panel **(A)** shows the genome of *Wolbachia* of *Anopheles moucheti* (*w*AnM) used in Figure 5B. Reads that were used to generate the genome of *w*AnM were used to map against the genome. Note the overall even read depth across the genome. Panel **(B)** shows the genome of *Wolbachia* of *Drosophila simulans* strain Noumea (*w*No) used in Figure 5A, with the same reads used in panel **(A)** mapped against the genome. Note the drop in read depth in identified prophage regions, particularly in the region of the Type III *cytoplasmic incompatibility factor* gene pair. Panel **(C)** shows the genome of *Wolbachia* of *An. demeilloni* (*w*AnD) used in Figure 5C. Reads that were used to generate the genome of *w*AnD were used to map against this genome. Like panel **A**, note the overall even read depth across the whole genome. Panel **(D)** shows the genome of wNo, with the read dataset used in panel **(C)** mapped against this genome. Note the partial drop in read depth in identified prophage regions, similar to panel **(B)**.

Supplementary figure 2: Unrooted version of Figure 1 from the main manuscript.

Supplementary figure 3: Midpoint-rooted **(A)** and unrooted **(B)** versions of the single-copy orthologous tree for *Wolbachia* of supergroup B.

Supplementary figure 4: Arrangement of genes in the three identified prophage regions of *w*AnD, aligned with a line graph of read depths of the same regions. Colours correspond to broad gene categories as defined by the key on the right. Uninterrupted genes have a solid colour, and genes marked as pseudogenised by NCBI's Prokaryotic Genome Annotation Pipeline [2] have an additional overlay.

Supplementary figure 5: Codon-based alignment of the *cif*B gene from the intact pair found within *w*AnD. The alignment compares this gene to the nucleotide *cif*B genes from four of the closest related orthologues that are predicted to encode fully functional proteins as identified from [3]. Overlaid above the alignment is the amino acid sequence for the *w*AnD *cif*B gene.

Supplementary figure 6: Unrooted version of Figure 5 from the main manuscript.

Supplementary figure 7: BRIG image comparisons of the *w*No geonome, compared against the genomes of *w*AnD, *w*AnM, as well as the two closely related strains of *Drosophila mauritiana* *w*Ma and *w*Mau (two outer-most rings). Note how these two genomes also show partial deletions in their genomes, focused on prophage regions, similar to what is seen in *w*No, *w*AnD and *w*AnM.

Supplementary table 1: Full detail of all Wolbachia genomes used in analysis in this manuscript. The results of this analysis were used to generate the results of Table 1, and Figure 2A/2B.

The following supplementary files have been uploaded to an external repository

(Figshare, https://figshare.com/projects/Wolbachia_endosymbionts_in_two_Anopheles_species_indicates_independent_acquisitions_and_lack_of_prophage_elements/126533).

Supplementary file 1 (10.6084/m9.figshare.17012996):

Compressed .zip file containing whole genome alignment outputs from snippy [4] and gubbins [5], as well as the resultant tree generated using IQtree in Newick file format, used to create Figure 1 [6]. See methods for full details.

Supplementary file 2 (10.6084/m9.figshare.19130846):

Compressed .zip file containing fasta sequences for single-copy orthologues obtained from OrthoFinder [7] that were used as input for alignment, before being concatenated and used to build the single-copy orthologous tree. All of these resultant files are also included (concatenated alignment, treefile, etc.)

Supplementary file 3 (10.6084/m9.figshare.17013005):

Compressed .zip file containing the results from OrthoFinder used to generate Figure 2A.

Supplementary file 4 (10.6084/m9.figshare.17012999):

Compressed .zip file containing the results from OrthoFinder used to generate Figure 2B.

Supplementary file 5 (10.6084/m9.figshare.17012993):

Compressed .zip file containing concatenated cifA/cifB gene alignment outputs, following the methods of Martinez et al. [3]. Sequences were taken from Martinez et al. supplementary materials, with the cif gene sequences for wAnD and wAnM replaced with those identified during this study. The zip file also contains the resultant tree in Newick file format, used to create Figure 5.

Supplementary file 6 (10.6084/m9.figshare.19130849):

Compressed .zip file of the results for the codon-based alignment of the intact *w*AnD *cif*B gene against four other, closely related intact orthologues found in other *Wolbachia* strains. These homologues were found within *w*Bai (from *Drosophila baimaii*), *w*Ha (*D. simulans*), *w*Ara (*D. arawakana*), and *w*Nik (*D. nikananu*).

1. **Alikhan NF, Petty NK, Ben Zakour NL, Beatson SA**. BLAST Ring Image Generator (BRIG): Simple prokaryote genome comparisons. *BMC Genomics*;12. Epub ahead of print 2011. DOI:

10.1186/1471-2164-12-402.

2. **Tatusova T, Dicuccio M, Badretdin A, Chetvernin V, Nawrocki EP, *et al.*** NCBI prokaryotic genome annotation pipeline. *Nucleic Acids Res* 2016;44:6614–6624.

3. **Martinez J, Klasson L, Welch JJ, Jiggins FM**. Life and Death of Selfish Genes: Comparative Genomics Reveals the Dynamic Evolution of Cytoplasmic Incompatibility. *Mol Biol Evol*. Epub ahead of print 2020. DOI: 10.1093/molbev/msaa209.

4. **Seeman T**. Snippy: Fast bacterial variant calling from NGS reads. https://github.com/tseemann/snippy (2015).

5. **Croucher NJ, Page AJ, Connor TR, Delaney AJ, Keane JA, *et al.*** Rapid phylogenetic analysis of large samples of recombinant bacterial whole genome sequences using Gubbins. *Nucleic Acids Res* 2015;43:e15.

6. **Nguyen LT, Schmidt HA, Von Haeseler A, Minh BQ**. IQ-TREE: A fast and effective stochastic algorithm for estimating maximum-likelihood phylogenies. *Mol Biol Evol* 2015;32:268–274.

7. **Emms DM, Kelly S**. OrthoFinder: solving fundamental biases in whole genome comparisons dramatically improves orthogroup inference accuracy. *Genome Biol* 2015;16:157.

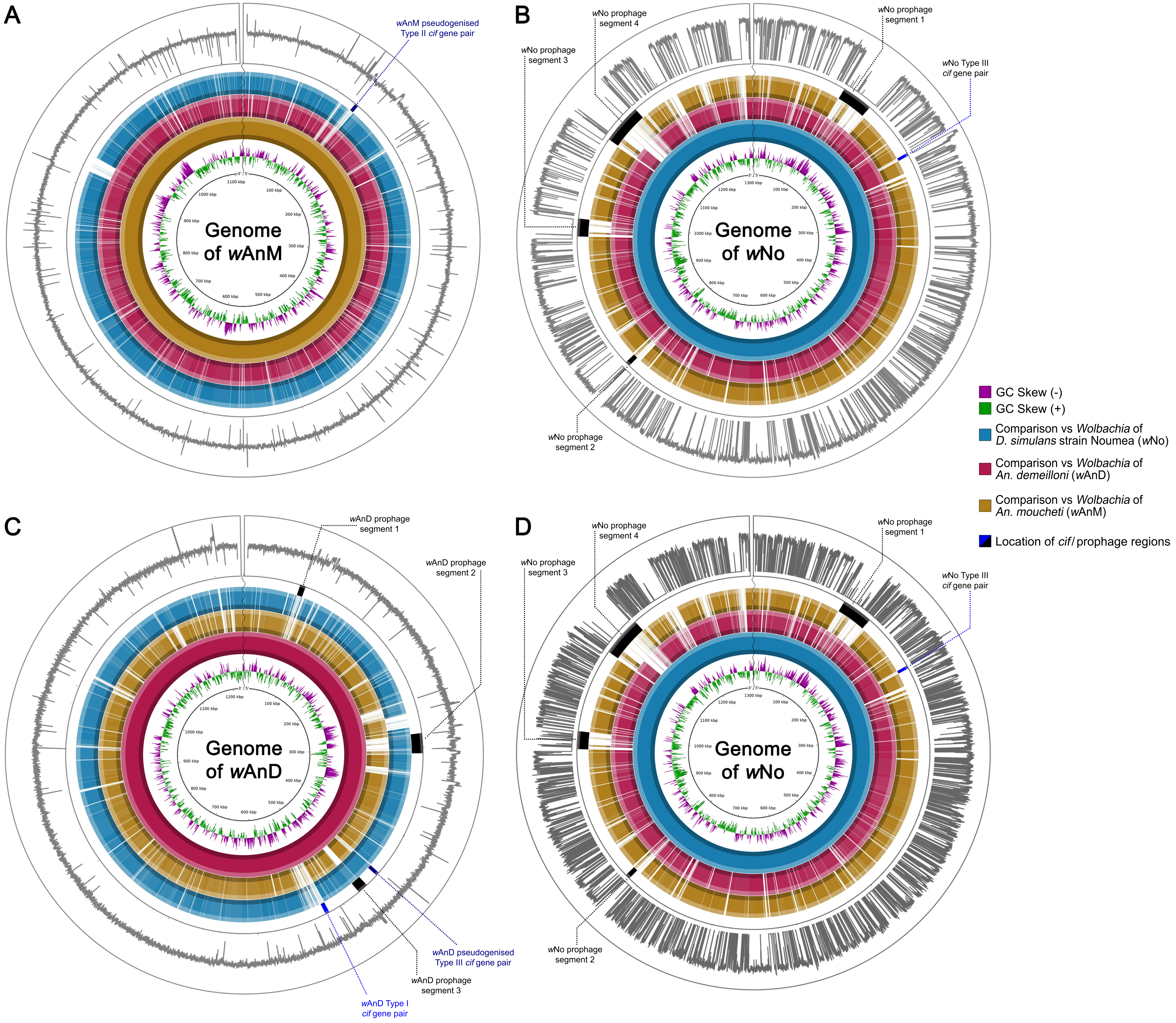
A
*w*AnM pseudogenised Type II *cif* gene pair
Genome of *w*AnM
B
*w*No prophage segment 4
*w*No prophage segment 1
*w*No prophage segment 3
*w*No Type III *cif* gene pair
Genome of *w*No
*w*No prophage segment 2
GC Skew (-)
GC Skew (+)
Comparison vs *Wolbachia* of *D. simulans* strain Noumea (*w*No)
Comparison vs *Wolbachia* of *An. demeilloni* (*w*AnD)
Comparison vs *Wolbachia* of *An. moucheti* (*w*AnM)
Location of *cif*/prophage regions
C
*w*AnD prophage segment 1
*w*AnD prophage segment 2
Genome of *w*AnD
*w*AnD pseudogenised Type III *cif* gene pair
*w*AnD prophage segment 3
*w*AnD Type I *cif* gene pair
D
*w*No prophage segment 4
*w*No prophage segment 1
*w*No prophage segment 3
*w*No Type III *cif* gene pair
Genome of *w*No
*w*No prophage segment 2

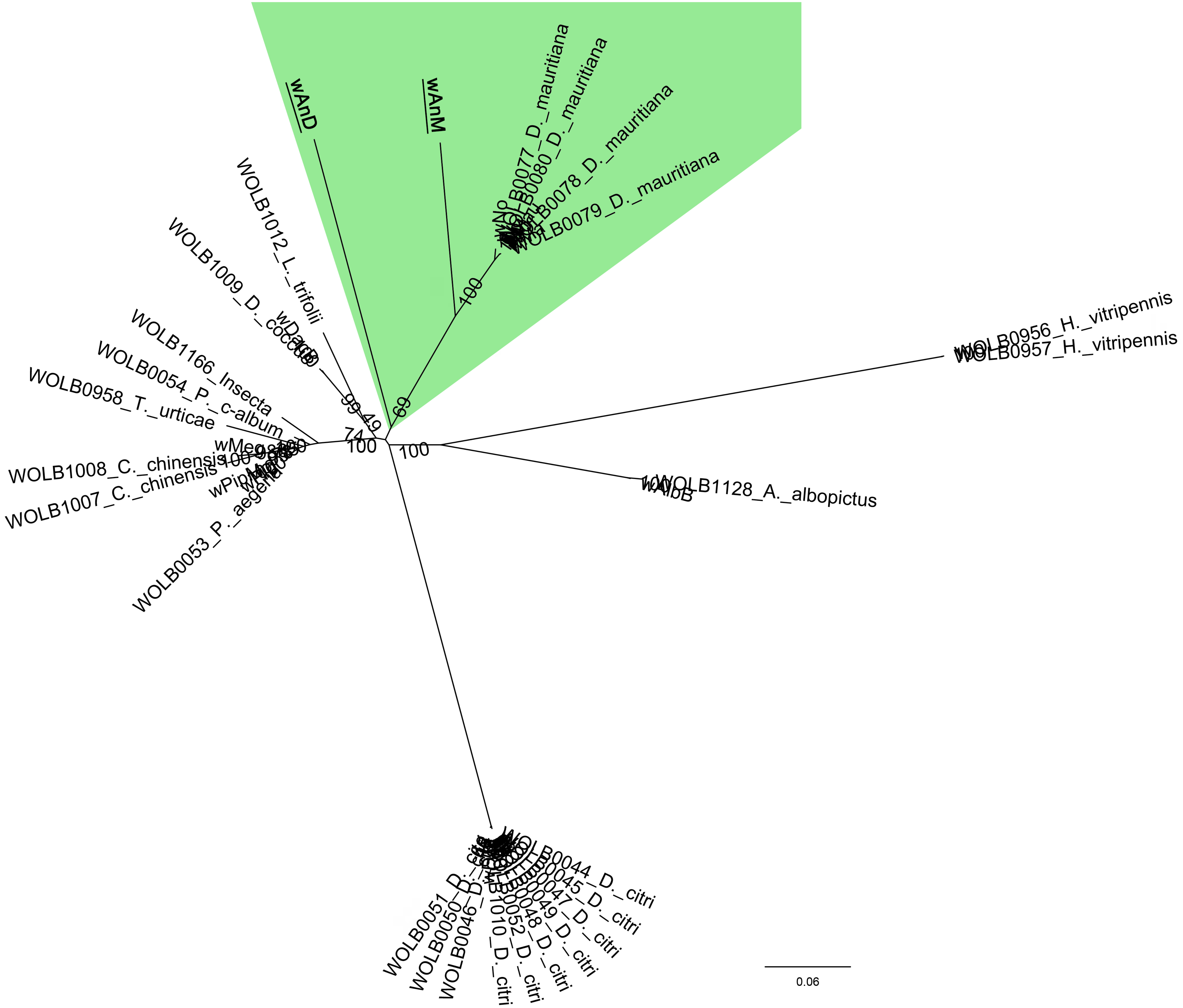

wAnD
wAnM
WOLB0077_D._mauritiana
WOLB0080_D._mauritiana
WOLB0078_D._mauritiana
WOLB0079_D._mauritiana
WOLB1012_L._trifolii
WOLB1166_Insecta
WOLB0054_P._c-album
WOLB0958_T._urticae
WOLB1008_C._chinensis
WOLB1007_C._chinensis
WOLB0053_P._aegeria
WOLB0956_H._vitripennis
WOLB0957_H._vitripennis
WOLB1128_A._albopictus
wAlbB
WOLB0044_D._citri
WOLB0045_D._citri
WOLB0047_D._citri
WOLB0049_D._citri
WOLB0048_D._citri
WOLB0052_D._citri
100
69
74
49
99
0.06

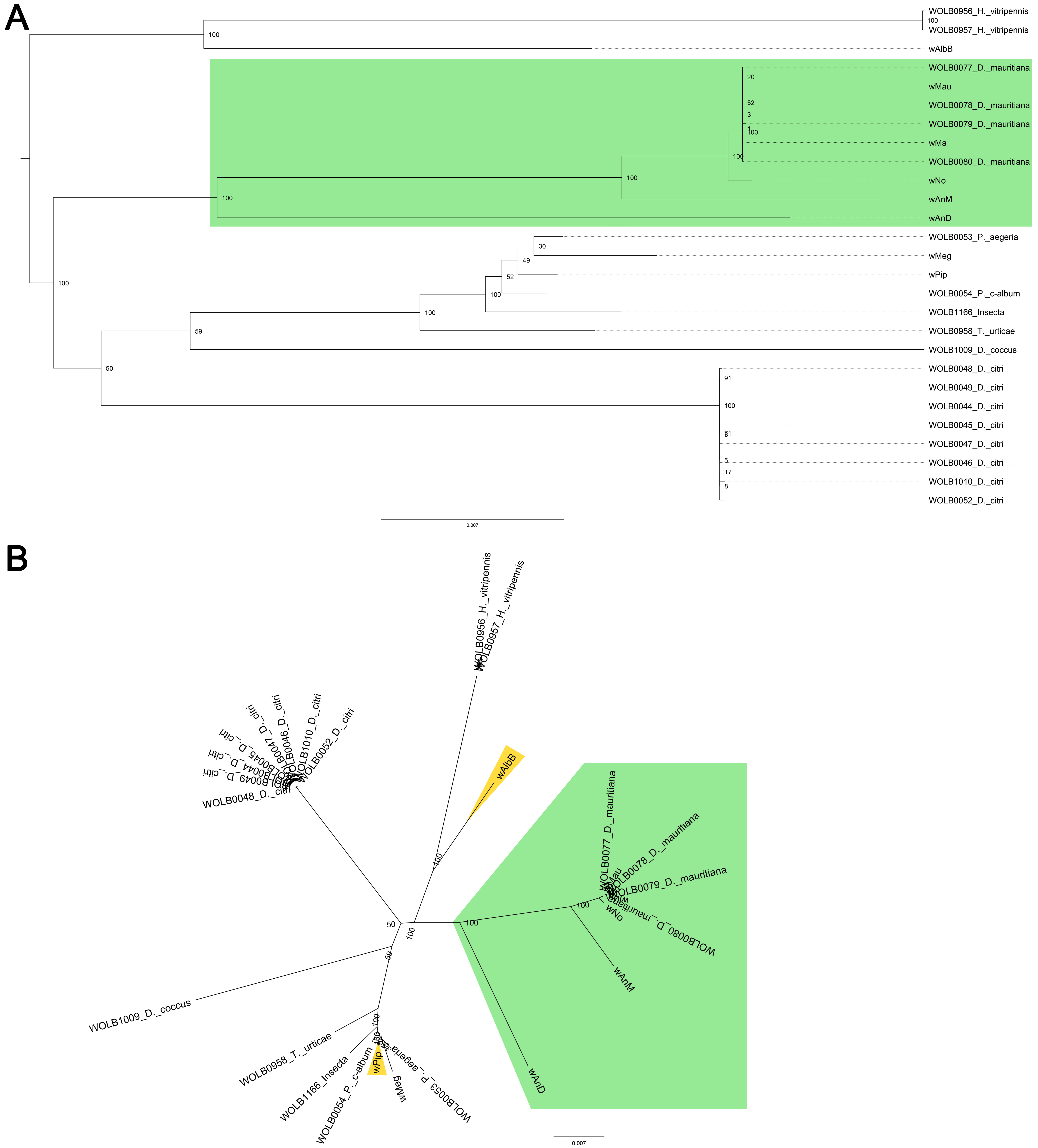
A
WOLB0956_H._vitripennis
WOLB0957_H._vitripennis
wAlbB
WOLB0077_D._mauritiana
wMau
WOLB0078_D._mauritiana
WOLB0079_D._mauritiana
wMa
WOLB0080_D._mauritiana
wNo
wAnM
wAnD
WOLB0053_P._aegeria
wMeg
wPip
WOLB0054_P._c-album
WOLB1166_Insecta
WOLB0958_T._urticae
WOLB1009_D._coccus
WOLB0048_D._citri
WOLB0049_D._citri
WOLB0044_D._citri
WOLB0045_D._citri
WOLB0047_D._citri
WOLB0046_D._citri
WOLB1010_D._citri
WOLB0052_D._citri
0.007
B
0.007

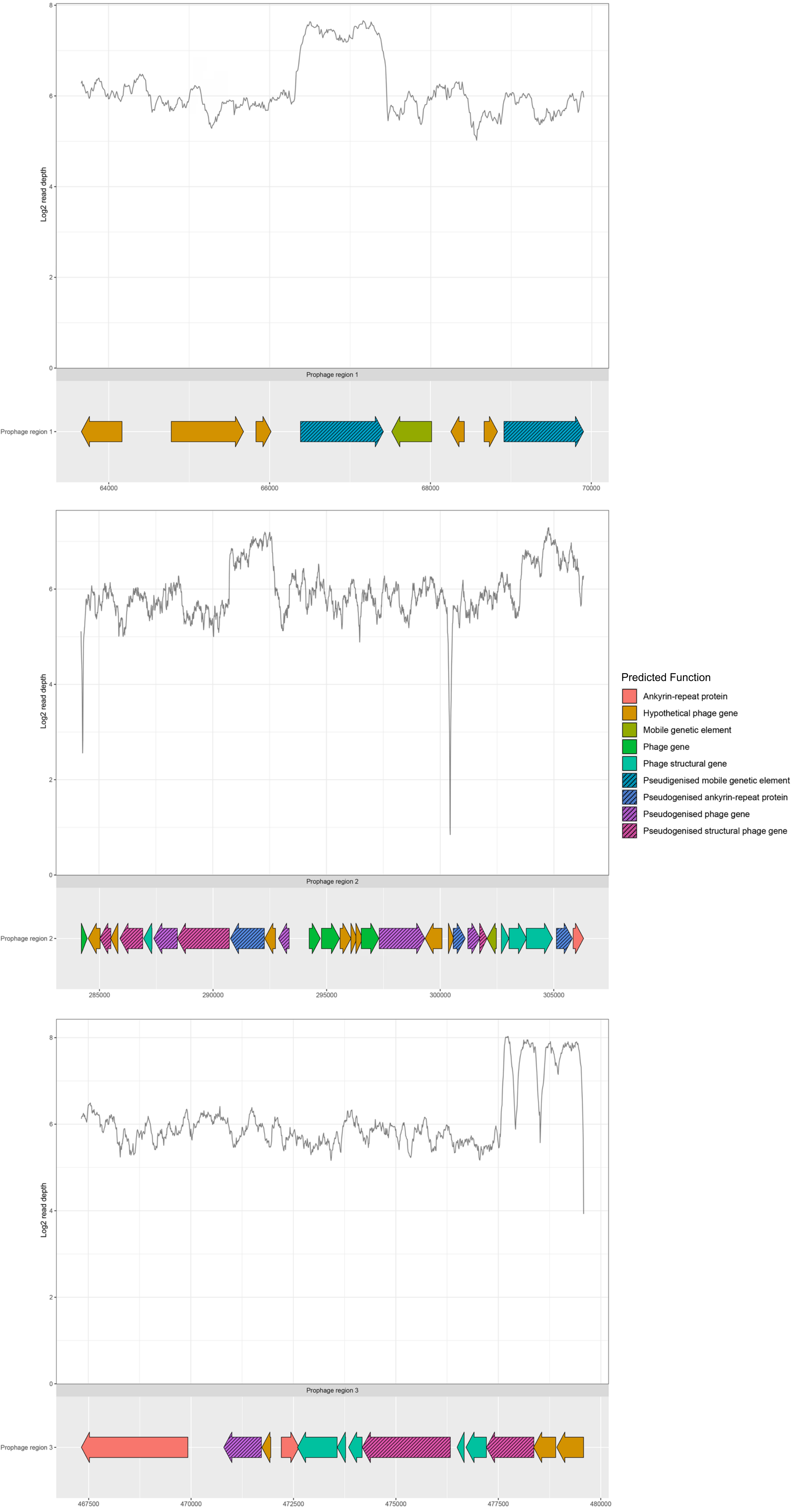

Log2 read depth
Prophage region 1
64000
66000
68000
70000
Prophage region 2
285000
290000
295000
300000
305000
Prophage region 3
467500
470000
472500
475000
477500
480000
Predicted Function
Ankyrin-repeat protein
Hypothetical phage gene
Mobile genetic element
Phage gene
Phage structural gene
Pseudigenised mobile genetic element
Pseudogenised ankyrin-repeat protein
Pseudogenised phage gene
Pseudogenised structural phage gene

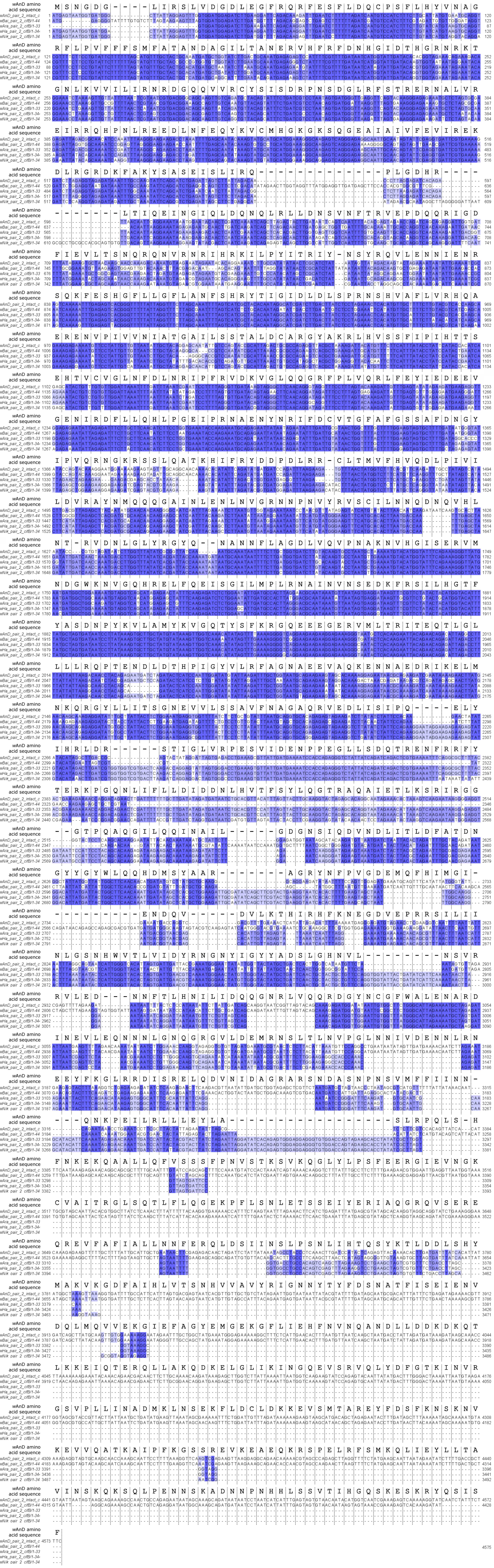

wAnD amino acid sequence
wAnD_pair_2_intact_c
wBai_pair_2_cifB/1-44
wBai_pair_2_cifB/1-33
wHa_pair_2_cifB/1-34-
wNik_pair_2_cifB/1-34

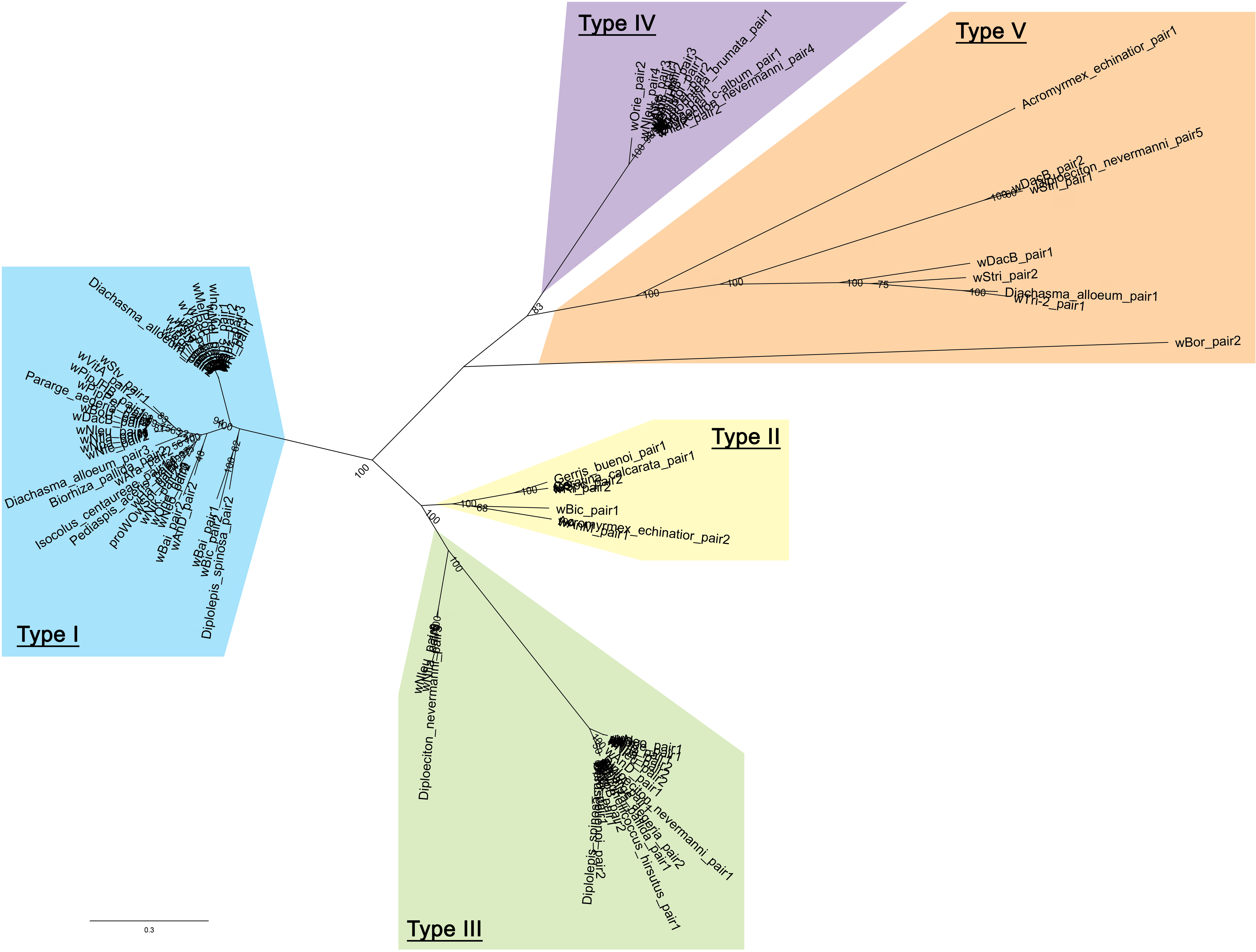

Type I
Type II
Type III
Type IV
Type V
Acromyrmex_echinatior_pair1
Diploeciton_nevermanni_pair5
wDacB_pair2
wStri_pair1
wDacB_pair1
wStri_pair2
Diachasma_alloeum_pair1
wTri-2_pair1
wBor_pair2
wOrie_pair2
Operophtera_brumata_pair1
Polygonia_c-album_pair1
Diploeciton_nevermanni_pair4
Gerris_buenoi_pair1
Ceratina_calcarata_pair1
wBic_pair1
Acromyrmex_echinatior_pair2
wAnM_pair1
Diachasma_alloeum_pair3
Biorhiza_pallida_pair2
Isocolus_centaureae
Pediaspis_aceris
Pararge_aegeria
wBai_pair1
wBic_pair2
Diplolepis_spinosa_pair2
Diploeciton_nevermanni_pair3
Diploeciton_nevermanni_pair1
Diplolepis_spinosa
0.3

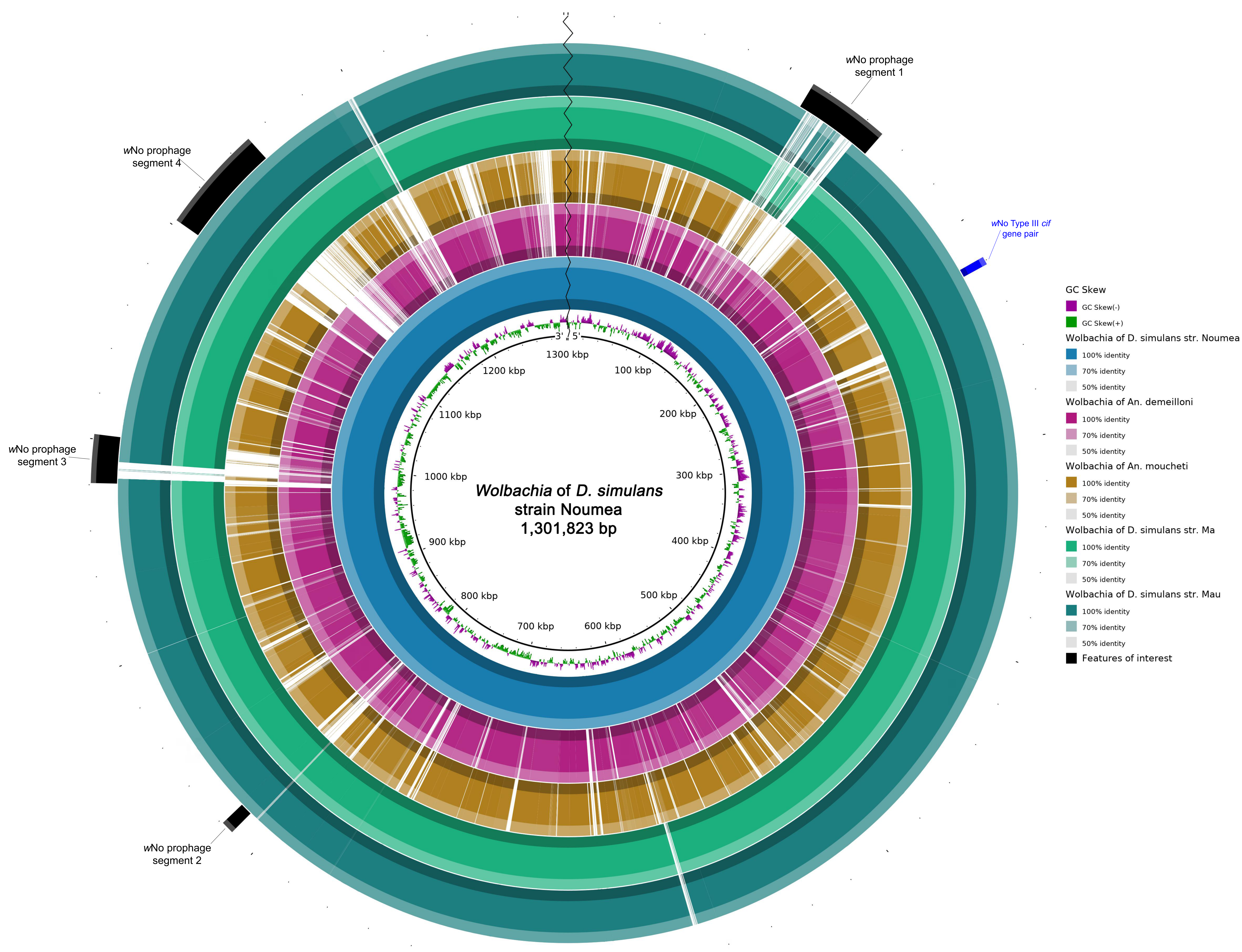

wNo prophage segment 1
wNo prophage segment 2
wNo prophage segment 3
wNo prophage segment 4
wNo Type III cif gene pair
3'
5'
1300 kbp
100 kbp
200 kbp
300 kbp
400 kbp
500 kbp
600 kbp
700 kbp
800 kbp
900 kbp
1000 kbp
1100 kbp
1200 kbp
Wolbachia of D. simulans strain Noumea 1,301,823 bp
GC Skew
GC Skew(-)
GC Skew(+)
Wolbachia of D. simulans str. Noumea
100% identity
70% identity
50% identity
Wolbachia of An. demeilloni
100% identity
70% identity
50% identity
Wolbachia of An. moucheti
100% identity
70% identity
50% identity
Wolbachia of D. simulans str. Ma
100% identity
70% identity
50% identity
Wolbachia of D. simulans str. Mau
100% identity
70% identity
50% identity
Features of interest
